# Supplementary material for: A Novel Electrokinetic-Based Technique for the Isolation of Circulating Tumor Cells
Source: Micromachines (Basel). 2023 Nov 5;14(11):2062. doi: 10.3390/mi14112062 (PMC10672846; doi:10.3390/mi14112062)
Supplement: Supplementary file 1 [file micromachines-14-02062-s001.zip › micromachines-2623429-supplementary.pdf]

Supplementary Materials

A Novel Electrokinetic-Based Technique for the Isolation of Circulating Tumor Cells

Mohammad K. D. Manshadi [1,*], Mahsa Saadat [2], Mehdi Mohammadi [3,4] and Amir Sanati Nezhad [4,5]

[1] Mechanical Engineering Department, Southern Methodist University, Dallas, TX 75206, USA
[2] Department of Biomedical Engineering, Florida International University, Miami, FL 33199, USA; msaad021@fiu.edu
[3] Department of Biological Sciences, University of Calgary, Calgary, AB T2N 1N4, Canada; mehdi.mohammadiashan@ucalgary.ca
[4] Department of Biomedical Engineering, Schulich School of Engineering, University of Calgary, Calgary, AB T2N 1N4, Canada; amir.sanatinezhad@ucalgary.ca
[5] BioMEMS and Bioinspired Microfluidic Laboratory, Department of Biomedical Engineering, Schulich School of Engineering, University of Calgary, Calgary, AB T2N 1N4, Canada
* Correspondence: mdehghanmanshadi@smu.edu

Table S1. Raw data corresponding to Figure 3B, Figure 3C, and Figure 3D.

**Fig. 3B**		**Fig. 3C**		Fig. 3D	
**x(μm)**	**y(μm)**	**x(μm)**	y(μm)	x(μm)	y(μm)
-300	75	-300	75	-300	75
-300	225	-300	225	-300	225
-288.569	225.0072	-283.719	225.0212	-276.981	225.0525
-288.569	74.9928	-283.719	74.9788	-276.981	74.9475
-277.141	225.0274	-267.447	225.068	-253.984	225.152
-277.141	74.9726	-267.447	74.932	-253.984	74.848
-265.716	225.0539	-251.179	225.1226	-230.995	225.261
-265.716	74.9461	-251.179	74.8774	-230.995	74.739
-254.293	225.0822	-234.916	225.179	-208.004	225.3613
-254.293	74.9178	-234.916	74.821	-208.004	74.6387
-242.87	225.1109	-218.652	74.7644	-185.013	74.5395
-242.87	74.8891	-218.652	225.2356	-185.013	225.4605
-231.449	225.1399	-202.389	74.7078	-162.022	74.4402
-231.449	74.8601	-202.389	225.2922	-162.022	225.5598
-220.027	74.8311	-186.145	74.7133	-139.09	74.5137
-220.027	225.1689	-186.145	225.2867	-139.09	225.4863
-208.605	74.8021	-169.91	225.2546	-116.185	225.3389
-208.605	225.1979	-169.91	74.7454	-116.185	74.6611
-197.184	74.7731	-153.675	225.2226	-93.5136	75.4465
-197.184	225.2269	-153.675	74.7774	-93.5136	224.5535
-185.775	74.7796	-137.44	225.1905	-71.2021	77.3751
-185.775	225.2204	-137.44	74.8095	-71.2021	222.6249
-174.376	225.185	-121.204	225.1584	-49.3505	81.2414
-174.376	74.815	-121.204	74.8416	-49.3505	218.7586
-162.977	225.1496	-104.969	225.1264	-27.6626	89.4261
-162.977	74.8504	-104.969	74.8736	-27.6626	210.5739
-151.578	225.1141	-88.7996	224.9196	-2.6779	104.9979
-151.578	74.8859	-88.7996	75.0804	-2.6779	195.0021
-140.18	225.0787	-72.9105	223.9395	45.7783	131.1788

-140.18	74.9213	-72.9105	76.0605	45.7783	168.8212
-128.781	225.0433	-57.2448	77.7822	281.8573	277.0764
-128.781	74.9567	-57.2448	222.2178	281.9247	22.9377
-117.382	225.0078	-41.8372	80.6969	67.5227	256.311
-117.382	74.9922	-41.8372	219.3031	67.6743	43.3926
-105.984	224.9724	-26.5556	85.4293	88.6637	124.0771
-105.984	75.0276	-26.5556	214.5707	89.9537	175.3731
-94.5849	224.937	-10.764	92.9142	116.8298	276.4132
-94.5849	75.063	-10.764	207.0858	119.37	22.46
-83.1862	224.9015	7.5343	104.2372	95.4993	121.1265
-83.1862	75.0985	7.5343	195.7628	100.368	176.7425
-71.9651	224.392	34.6425	120.4676	91.4389	254.3672
-71.9651	75.608	34.6425	179.5324	95.7635	40.9285
-60.8395	223.598	100.1628	140.1668	215.7634	46.7019
-60.8395	76.402	100.1628	159.8332	263.2583	263.0353
-49.82	222.4412	280.607	261.8603	98.2115	111.0051
-49.82	77.5588	280.6311	38.1524	114.0417	180.1055
-38.9187	79.2197	254.8287	295.8971	88.2627	216.6477
-38.9187	220.7803	255.5522	4.1101	88.7192	68.8682
-28.1172	81.555	232.7631	298.9205	99.8911	243.8031
-28.1172	218.445	234.9765	1.0914	117.2884	38.6401
-17.3411	84.7882	261.4417	299.67	117.6609	258.3557
-17.3411	215.2118	264.4198	0.3408	158.3031	21.5869
-6.3928	89.1911	307.3954	299.8469	124.0372	260.8102
-6.3928	210.8089	310.6746	0.1575	180.8421	17.3155
5.1501	95.0783	357.201	299.9077	116.4369	252.4284
5.1501	204.9217	360.681	0.0936	163.4246	22.0185
18.1981	102.8238	405.9934	299.9183	106.389	234.3477
18.1981	197.1762	409.1568	0.0863	131.196	37.2393
34.6515	112.7173	445.09	299.7796	108.9134	208.8279
34.6515	187.2827	447.6738	0.2521	109.5114	61.9546
58.9265	124.7229	477.0209	299.3761	111.3798	93.0278
58.9265	175.2771	479.5074	0.6884	143.3872	185.6879
104.3753	137.3151	504.6672	298.9122	156.4086	114.7466
104.3753	162.6849	507.0345	1.1643	224.8987	258.0332
187.6708	134.2045	528.578	298.5422	142.1059	264.697
187.6708	165.7955	530.7973	1.5353	239.1319	27.5852
288.038	269.5889	549.7672	298.2793	116.6674	209.0743
288.0542	30.4184	551.8646	1.7964	120.4285	55.3598
327.738	291.903	569.1089	298.1012	138.4974	108.0261
327.8563	8.0981	571.1187	1.9725	190.3846	215.7262
446.1504	292.9198	587.2578	297.9828	127.7414	249.3406
446.399	7.1106	589.2035	2.0891	216.9524	20.8679
513.3922	284.1382	604.6268	297.9061	120.4267	87.1044
513.5322	15.9068	606.5294	2.1644	158.3032	191.4242
548.7057	277.2815	621.5194	297.8562	137.2294	255.2011
548.8133	22.7738	623.3918	2.2133	232.3934	29.85
573.2871	272.0158	638.0965	297.825	129.2084	93.6579
573.3785	28.047	639.9493	2.2438	177.2836	201.7168
592.6587	268.0238	654.4927	297.805	125.4177	220.8413
592.7403	32.0446	656.3329	2.2634	156.077	34.3199

609.1029	265.0459	670.7639	297.793	162.6716	267.7278
609.1783	35.0267	672.5955	2.2751	189.0855	78.0977
623.7952	262.8485	686.9662	297.7856	147.8902	98.7836
623.8664	37.2272	688.7923	2.2822	217.3012	260.3494
637.3567	261.2519	703.12	297.7817	133.6275	83.4176
637.4252	38.8258	704.9429	2.286	175.446	207.0194
650.1801	260.1088	719.2556	297.7791	133.4736	72.3746
650.2467	39.9703	721.0762	2.2885	163.8056	203.4868
662.5074	259.3022	735.3764	297.7776	135.4149	70.2283
662.5727	40.7778	737.1956	2.2899	165.3476	205.4147
674.4985	258.7444	751.4892	297.7769	137.5853	76.5568
674.563	41.3363	753.3076	2.2907	177.4689	213.2818
686.2663	258.364	767.5966	297.7766	146.1027	87.4205
686.3305	41.7171	769.4147	2.2909	208.9389	259.0496
697.8841	258.1107	783.7041	297.7763	168.3315	90.2796
697.9481	41.9707	785.5219	2.2912	180.6849	269.6038
709.4038	257.9454	799.8096	297.7762	143.3905	240.0411
709.4677	42.1361	801.6274	2.2913	213.0198	37.8766
720.8559	257.845	815.9149	297.7762	149.688	213.8313
720.9198	42.2367	817.7326	2.2913	162.3656	39.4235
732.2691	257.7849	832.02	297.7762	142.088	73.4026
732.3331	42.2968	833.8378	2.2913	185.3527	226.4994
743.6558	257.7552	848.1251	297.7762	165.8494	88.9922
743.7198	42.3265	849.943	2.2913	179.1323	268.3224
755.0317	257.7399	864.2301	297.7762	142.4851	231.1947
755.0958	42.3418	866.0482	2.2913	213.4709	31.3905
766.4003	257.7364	880.3352	297.7762	149.0015	52.3843
766.4646	42.3452	882.1534	2.2913	163.6498	211.0757
777.7667	257.7395	896.4402	297.7761	149.423	84.7243
777.8311	42.3422	898.2585	2.2914	213.5135	268.2208
789.1335	257.746	912.5452	297.7761	148.4565	246.5588
789.198	42.3357	914.3637	2.2914	188.2732	68.1395
800.5022	257.7539	928.6501	297.7761	150.973	214.3599
800.5668	42.3277	930.4689	2.2914	169.9268	35.68
811.8735	257.7621	944.7551	297.7761	143.0755	73.9435
811.9383	42.3195	946.574	2.2914	192.3818	239.9133
823.2478	257.7698	960.8601	297.7761	163.9428	260.8623
823.3126	42.3118	962.6792	2.2914	170.9411	86.8135
834.6247	257.7767	976.9649	297.7761	144.1208	222.8654
834.6897	42.3049	978.7842	2.2914	201.8833	28.1372
846.0043	257.7825	993.0697	297.7761	144.4128	60.9995
846.0693	42.299	994.8892	2.2914	175.8277	216.2444
857.3861	257.7874	1009.175	297.7761	156.9899	88.2722
857.4512	42.2941	1010.994	2.2914	190.999	271.0998
868.7698	257.7914	1025.279	297.7761	143.1192	234.1684
868.835	42.2901	1027.099	2.2914	210.2129	39.0063
880.1549	257.7946	1041.384	297.7761	153.9798	47.0034
880.2203	42.2869	1043.204	2.2914	161.9368	211.1713
891.5418	257.7968	1057.489	297.7761	147.3331	82.4081
891.6072	42.2847	1059.309	2.2914	214.0955	267.0775
902.9289	257.7989	1073.594	297.7761	149.1565	247.474

902.9944	42.2826	1075.414	2.2914	184.57	74.1162
914.3172	257.8004	1089.698	297.7761	150.6254	214.6822
914.3828	42.2811	1091.519	2.2914	174.6781	33.5466
925.7059	257.8015	1105.803	297.7761	142.8751	72.27
925.7716	42.28	1107.624	2.2914	191.8404	238.8303
937.0953	257.8023	1121.908	297.7761	164.3681	261.1145
937.1611	42.2793	1123.729	2.2914	169.1646	87.5135
948.4851	257.8027	1138.013	297.7761	144.1244	222.9494
948.551	42.2788	1139.834	2.2914	204.8583	28.3965
959.875	257.8032	1154.117	297.7761	144.9649	59.7187
959.9409	42.2783	1155.939	2.2914	175.9691	216.3991
971.2648	257.8036	1170.222	297.7761	156.0486	87.9839
971.3308	42.2779	1172.044	2.2914	190.5364	271.0174
982.6548	257.8039	1186.327	297.7761	143.0962	233.9147
982.721	42.2776	1188.149	2.2914	207.7007	40.4966
994.0452	257.804	1202.432	297.7761	155.239	45.7972
994.1114	42.2775	1204.254	2.2914	162.3345	211.2035
1005.436	257.8041	1218.536	297.7761	146.7407	81.679
1005.502	42.2774	1220.359	2.2914	213.7602	267.677
1016.826	257.8041	1234.641	297.7761	148.5004	246.5037
1016.892	42.2774	1236.464	2.2914	183.2518	75.9699
1028.216	257.8042	1250.746	297.7761	151.4334	214.1602
1028.283	42.2773	1252.568	2.2914	176.9105	32.6509
1039.607	257.8042	1266.851	297.7761	142.7612	71.2793
1039.673	42.2773	1268.673	2.2914	193.6189	242.4369
1050.997	257.8042	1282.955	297.7761	162.5062	259.9172
1051.064	42.2773	1284.778	2.2914	167.783	87.9953
1062.388	257.8042	1299.06	297.7761	144.4279	222.1231
1062.455	42.2773	1300.883	2.2914	207.7633	28.8996
1073.778	257.8042	1315.165	297.7761	145.7375	58.0039
1073.845	42.2773	1316.988	2.2914	176.9178	217.1271
1085.169	257.8042	1331.27	297.7761	154.4551	87.4408
1085.236	42.2773	1333.093	2.2914	189.0613	270.7672
1096.559	257.8042	1347.374	297.7761	142.9879	233.5875
1096.626	42.2773	1349.198	2.2914	201.7922	43.8726
1107.95	257.8042	1363.479	297.7761	157.5537	43.7699
1108.017	42.2773	1365.303	2.2914	162.3848	211.1552
1119.34	257.8042	1379.584	297.7761	145.7985	80.3989
1119.407	42.2773	1381.408	2.2914	213.9027	267.5103
1130.731	257.8042	1395.689	297.7761	148.7749	246.9524
1130.798	42.2773	1397.513	2.2914	180.94	78.8698
1142.121	257.8042	1411.793	297.7761	150.891	214.4572
1142.189	42.2773	1413.618	2.2914	181.024	31.2443
1153.512	257.8042	1427.898	297.7761	142.7482	69.6978
1153.579	42.2773	1429.723	2.2914	192.3067	239.7716
1164.902	257.8042	1444.003	297.7761	163.968	260.8835
1164.97	42.2773	1445.828	2.2914	166.1536	88.3753
1176.293	257.8042	1460.107	297.7761	144.066	222.9207
1176.36	42.2773	1461.933	2.2914	209.9328	29.5973
1187.683	257.8041	1476.212	297.7761	146.3503	56.9087
1187.751	42.2774	1478.037	2.2914	175.5155	215.9743

1199.074	257.8041	1492.317	297.7761	153.7557	87.1257
1199.141	42.2774	1494.142	2.2914	192.0931	271.275
1210.464	257.8041			143.2034	234.7375
1210.532	42.2774			199.5033	46.1198
1221.855	257.8041			158.3985	43.0894
1221.923	42.2774			161.133	211.1494
1233.245	257.8041			145.5118	79.9439
1233.313	42.2774			214.2924	266.1781
1244.636	257.8041			149.928	248.5011
1244.704	42.2774			180.1487	79.755
1256.026	257.8041			149.8258	215.2162
1256.094	42.2774			182.4581	30.8071
1267.417	257.8041			142.7247	69.0221
1267.485	42.2774			189.9438	235.0471
1278.807	257.8041			165.1496	88.5917
1278.875	42.2774			166.5451	262.4333
1290.197	257.8041			143.6567	224.0796
1290.266	42.2774			211.4891	30.2937
1301.588	257.8041			147.0315	55.6844
1301.657	42.2774			173.8851	214.8338
1312.978	257.8041				
1313.047	42.2774				
1324.369	257.8041				
1324.438	42.2774				
1335.759	257.8041				
1335.828	42.2774				
1347.15	257.8041				
1347.219	42.2774				
1358.54	257.8041				
1358.609	42.2774				
1369.931	257.8041				
1370	42.2774				
1381.321	257.8041				
1381.39	42.2774				
1392.712	257.8041				
1392.781	42.2774				
1404.102	257.8041				
1404.172	42.2774				
1415.493	257.8041				
1415.562	42.2774				
1426.883	257.8042				
1426.953	42.2773				
1438.274	257.8042				
1438.343	42.2773				
1449.664	257.8042				
1449.734	42.2773				
1461.054	257.8042				
1461.124	42.2773				
1472.445	257.8043				
1472.515	42.2772				
1483.835	257.8043				

1483.905	42.2772
1495.226	257.8043
1495.296	42.2772

Table S2. Raw data corresponding to Figure 4A.

Electric field (V/cm)	dp=5	dp=10	dp=15
35	0	0	0
45	0	0	11
55	0	2	20
65	0	12	59
75	0	65	96
80	6	85	100
85	35	93	100
90	91	100	100
95	100	100	100

Table S3. Raw data corresponding to Figure 4B.

Particle Diameter	Trapped 85	75	65
1	0	0	0
2	0	0	0
3	0	0	0
4	3	1	0
5	29	3	0
6	72	5	0
7	90	16	2
8	92	26	4
10	94	47	10
12	97	76	24
14	99	91	44
15	100	94	57
17	100	97	81
20	100	100	94
25	100	100	100

Table S4. Raw data corresponding to Figure 5E.

zeta	trap
10	100
30	100
50	90
70	30
100	8
